# Use of lithium in clozapine-induced neutropenia: a case report

**DOI:** 10.1186/1756-0500-7-635

**Published:** 2014-09-12

**Authors:** Chathurie Suraweera, Raveen Hanwella, Varuni de Silva

**Affiliations:** University Psychological Medicine Unit, National Hospital of Sri Lanka, Colombo, Sri Lanka; Department of Psychological Medicine, Faculty of Medicine, Kynsey Road, Colombo 08, Sri Lanka

**Keywords:** Neutropenia, Clozapine, Lithium carbonate

## Abstract

**Background:**

The literature describing the long-term use of lithium carbonate to reinstate reduced levels of white blood cell counts in patients treated with clozapine is scarce. We describe a case of successful recommencement of clozapine on a patient who developed risk level of neutropenia which was corrected by lithium carbonate. He was followed up for a period of one year.

**Case presentation:**

We report a 40-year-old Sri Lankan male who developed neutropenia and low white blood cell counts following commencement of clozapine. We were successful in restarting clozapine after the addition of lithium carbonate to increase the cell counts. Clozapine was increased to 700 mg a day with 500 mg of lithium carbonate. The patient remains stable after one year with no further episodes of neutropenia.

**Conclusion:**

Lithium carbonate can successfully be used to treat clozapine-induced neutropenia.

## Background

It is estimated that approximately 30% of patients with schizophrenia are resistant to typical and atypical antipsychotics and warrant treatment with clozapine. Clozapine should be offered to patients with treatment resistant schizophrenia (TRS) as it is the antipsychotic with most robust evidence for improving psychopathology and the quality of life [1]. However, clozapine carries a 0.9% risk of causing agranulocytosis and 2.7% risk of neutropenia [2] which could be fatal. Over 80% of such cases are seen within the first 18 weeks of treatment. Some patients who develop agranulocytosis may be genetically predisposed [3].

As a result, most clinicians are reluctant to initiate clozapine on patients. Lithium carbonate has been known to increase the white blood cell (WBC) counts in patients with leucocytopenia due to oncological causes. This has prompted clinicians to explore the possibility of using lithium carbonate in clozapine-induced neutropenia.

## Case presentation

Our patient is a 40-year-old Sri Lankan male who was diagnosed with paranoid schizophrenia at the age of 22 years and was resistant to adequate trials of trifluoperazine, olanzapine and risperidone. Therefore, he was started on clozapine. The patient had delusions of reference, delusional perception and commanding hallucinations which affected his day to day functioning to a great degree. He lost his job as a result of ongoing psychopathology.

His baseline white cell counts were only marginally higher than the levels recommended before initiating treatment with clozapine. The effect of clozapine and lithium carbonate on the leucocyte count of the patient is given in Table 1.

**Table 1:** **Effect of clozapine and lithium carbonate on white blood cell and neutrophil counts**

Day	0	1	7	9	10	11	13	14	16	20	27	34
**Total dose of clozapine (mg)**		12.5	50	62.5	0			12.5		50	75	150
**Dose of lithium carbonate (mg)**					250	500	500	500	500	500	500	500
**White cell count (10** ^**3**^ **/μl)**	6.03		5.8	4.9				6.3	6.2	7.4	7.8	8.3
**Neutrophil count (10** ^**3**^ **/μl)**	2.82		2.4	2.0				2.6	3.4	3.7	3.7	3.0

In order to initiate clozapine, patients must have a baseline WBC count of 4.0 ×10^3^/μl and a neutrophil count of 2.5 × 10^3^/μl. Clozapine must be withheld if the WBC count drops below the ‘red’ cut-off of 3.0 × 10^3^/μl or the neutrophil count falls below 1.5 × 10^3^/μl.

In patients with benign ethnic neutropenia, the neutrophil count before commencement of clozapine is low and persistently tends to be around the risk level. Our patient had a low baseline WBC count of 6.02 × 10^3^/μl and a neutrophil count of 2.82 × 10^3^/μl before initiation of clozapine. The WBC and neutrophil counts decreased to 4.9 × 10^3^/μl and 2.0 × 10^3^/μl respectively, at a clozapine dose of 62.5 mg on day 9. In such patients lack of exercise, being a non-smoker or simply having blood drawn at the wrong time of day could result in clozapine treatment having to be stopped resulting in negative consequences for the patient [4]. Approximately 90% of WBC remains in storage in the bone marrow. The total lifespan of a neutrophil is 11-14 days but they die within hours after entering the circulation. Infection stimulates release and can triple the WBC count in a matter of hours. Neutrophils can either circulate freely in the bloodstream or marginate after being released from the bone marrow.

Lithium increases the neutrophil count and total WBC count both acutely and chronically [5]. This ‘side-effect’ of lithium has been used successfully to raise the WBC during cancer chemotherapy and in patients treated with carbamazepine. The mechanism by which lithium increases neutrophil count is not completely understood and the effect is poorly quantified. Neutrophilia does not seem to be clearly dose related although a minimum lithium serum level of 0.4 mmol/l may be required. A mean neutrophil count of 11.9 × 10^3^/μl with a mean rise of 2.6 × 10^3^/μls has been reported in patients treated with lithium [6, 7].

**Figure 1:**
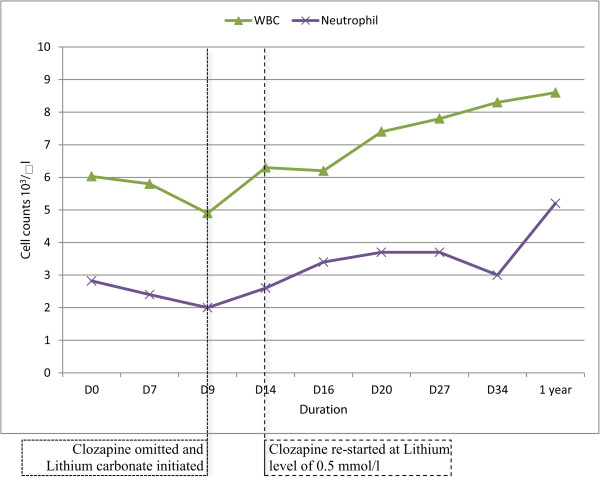
**Effect of clozapine and lithium carbonate on white blood cell and neutrophil counts.** The white blood cell and neutrophil counts dropped to 4.9 × 10^3^/μl and 2.0 × 10^3^/μl respectively at a clozapine dose of 62.5mg/d. It increased to 6.3 × 10^3^/μl and 2.6 ×10^3^/μl after addition of lithium carbonate.

We restarted clozapine at a lithium level of 0.5 mmol/l on day 14 in our patient when he had a neutrophil count of 2.6 × 10^3^/μl and a WBC count of 6.3 × 10^3^/μl. He had a mean neutrophil count of 5.16 × 10^3^/μl and a mean WBC count of 10.14 × 10^3^/μl 6 months after initiation of lithium. At 1 year he had a WBC count of 8.6 × 10^3^/μl and a neutrophil count of 5.2 × 10^3^/μl. His mean lithium level at the end of 6 months was 0.55mmol/l. and 0.43 mmol/l at the end of one year (Figure 1).

## Conclusion

Our patient remains well and is currently employed. However, there are potential risks that should be borne in mind. Re-challenge with clozapine have faced with successes [8] and failures. In a retrospective review of 53 cases of clozapine re-challenge in the United Kingdom and Ireland, 62% of the patients did not develop a blood dyscrasia [9]. In 85% of the cases that developed a blood dyscrasia on re-challenge, it occurred sooner, lasted longer and was more severe than with the initial trial of clozapine. Therefore, when deciding on re-challenge with clozapine, clinicians must carefully weigh the risks and benefits. Not only the possibility of another episode of neutropenia, but toxic effects of lithium and other side effects of clozapine need particular attention.

## Consent

Written informed consent was obtained from the patient for publication of this case report. A copy of the written consent is available for review by the Editor-in-Chief of this journal.

## References

[1] Kane JM (1992). Clinical efficacy of clozapine in treatment-refractory schizophrenia: an overview. Br J Psychiatry Suppl.

[2] Lieberman JA (1998). Maximizing clozapine therapy: managing side effects. J Clin Psychiatry.

[3] Munro J, O’sullivan D, Andrews C, Arana A, Mortimer A, Kerwin R (1999). Active monitoring of 12,760 clozapine recipients in the UK and Ireland. Beyond pharmacovigilance. Br J Psychiatry.

[4] Abramson N, Melton B (2000). Leukocytosis: basics of clinical assessment. Am Fam Physician.

[5] Lapierre G, Stewart RB (1980). Lithium carbonate and leukocytosis. Am J Hosp Pharm.

[6] Blier P, Slater S, Measham T, Koch M, Wiviott G (1998). Lithium and clozapine-induced neutropenia/agranulocytosis. Int Clin Psychopharmacol.

[7] Paton C, Esop R (2005). Managing clozapine-induced neutropenia with lithium. Psychiatr Bull.

[8] Ghaznavi S, Nakic M, Rao P, Jian H, Brewer JA, Hannestad J, Bhagwagar Z (2008). Rechallenging with clozapine following neutropenia: treatment options for refractory schizophrenia. Am J Psychiatry.

[9] Atkin K, Kendall F, Gould D, Freeman H, Liberman J, O Sullivan D (1996). Neutropenia and agranulocytosis in patients receiving clozapine in the UK and Ireland. Br J Psychiatr.

